# Supplementary material for: The Performance of Lateral Flow Tests in the Age of the Omicron: A Rapid Systematic Review
Source: Life (Basel). 2022 Nov 21;12(11):1941. doi: 10.3390/life12111941 (PMC9695766; doi:10.3390/life12111941)
Supplement: Supplementary file 1 [file life-12-01941-s001.zip › life-1960103-supplementary.pdf]

Table S1. Risk of bias of studies included in this reviewe, assessed using Meta Analysis of Statistics Assessment and Review Instrument (MAStARI) appraisal tool

Study	Random sample	Criteria for inclusion	Confounding factors	Outcomes (objective crtieria)	Sufficient description of the groups	Follow up	Outcomes of people who withdrew	Outcomes (measured in a reliable way)	Appropriate statistical analysis
Adamson, 2022 [13]	-	-	-	+	-	+	-	?	+
Bayart, 2022 [14]	-	-	?	+	-	-	-	+	+
Bekliz, 2022 [15]	-	-	?	+	-	-	-	+	+
Deerain, 2021 [10]	NA	NA	NA	+	NA	NA	NA	+	-
Gourgeon, 2022 [16]	-	-	-	+	-	-	-	?	-
Kanjilal, 2022 [17]	-	-	-	+	-	-	-	?	-
Schrom, 2022 [18]	-	-	-	+	-	-	-	?	-
Stanley, 2022 [19]	NA	NA	NA	+	NA	NA	NA	+	-
Tsao, 2022 [20]	-	-	-	+	-	-	-	?	-
Weishampel, 2022 [21]	NA	NA	NA	+	NA	NA	NA	+	-

Legend: +, low risk of bias; -, high risk of bias; ?, unclear; NA, not applicable
